# The implementation process of decentralization of health services: a qualitative perspective from Sudan

**DOI:** 10.3389/fpubh.2024.1439219

**Published:** 2024-11-08

**Authors:** Bandar Noory, Sara Hassanain, Kassem Kassak

**Affiliations:** ^1^Department of Epidemiology and Population Health, Faculty of Health Sciences, American University of Beirut, Beirut, Lebanon; ^2^Independent Consultant, Khartoum, Sudan; ^3^Department of Health Management and Policy, Faculty of Health Sciences, American University of Beirut, Beirut, Lebanon

**Keywords:** decentralization, process of implementation, health system, governance of health services, stakeholder involvement, conflict of interest

## Abstract

**Background:**

Health system reform initiatives have increasingly embraced decentralization as a key trend. The implementation process and its outcomes are influenced by a myriad of factors, including economic forces, political dynamics, and ideological factors. Comprehending and carefully examining the implementation phase of decentralization and its consequences to achieve desired outcomes is crucial. Notably, this phase is often considered the weakest aspect of policy reforms, especially in developing countries. Therefore, this study aimed to explore and analyze the implementation phase of decentralization and its implications as essentials for achieving intended goals. The study objectives evolved around examining the entire decentralization as a multifaceted social and political process in Khartoum State, Sudan, from its early stages of decision-making, policy formulation, and implementation process and the influencing contextual factors.

**Methods:**

This study was an exploratory qualitative study that employed in-depth interviews to gather perspectives of healthcare providers and policymakers, semi-structured observations, and thematic analysis. The study utilized Grindle’s framework for analysis.

**Findings:**

This study revealed a political nature of decision-making, with a top–down approach to the implementation, which also lacked stakeholder involvement. It showed a lack of transparency, official documentation, and proper handover procedures from the Ministry of Health to the devolved hospitals during the implementation process. A conflict of interest between the federal and the state level was also reported. It is important to note that this process occurred within the context of structural adjustment program (SAP) schemes, which had already empowered the private sector in Sudan.

**Conclusion:**

This study documented the implementation process of decentralization of health services and its influencing factors. The study recommended reforming the decentralization policy through consultative stakeholder involvement and by implementing a concurrent responsibility paradigm that divides authority between the federal and state levels.

## Introduction

Decentralization has been implemented as part of health system reform initiatives in numerous developed and developing countries with the goal of enhancing access to care, promoting efficiency, equity, and quality, and increasing accountability (1–3). This global trend involves transferring the governing authority for planning or service delivery from central to local governments or from large to district facilities (35). Usually, the degree of authority transference also varies among countries, ranging from involvement of the community, devolution to local governments, de-concentration of operations, and authority transference to independent bodies outside the control of the health sector and government (4–7).

The influence of decentralization on health systems and their results is multifaceted and mixed, as it is shaped by a range of elements, such as ideology, social dynamics, and political circumstances. A systematic review revealed that decentralization effects are unpredictable and reflected mixed results and highlighted the necessity of examining each health system building block and the contextual factors (8). Ultimately, these also intersect with the intricate function of the health system, its connected building blocks, and its anticipated outcomes (7, 9). Mixed findings regarding the impact of decentralization on the health system performance and health outcomes have also been reported by another systematic review (10). Nevertheless, the process of decentralization of health services implementation in a local context is critical in determining the policy’s contents and sequential outcomes (3, 11, 36). Variations in policy outcomes can be attributed to several contextual factors in which the policy is being implemented and the implementation process, including the involvement of healthcare providers in decision-making and the stages of implementation, their espoused perception and behavior towards the process of policy and its outcome (12).

For instance, in some countries such as Ghana, Uganda, Kenya, and Tanzania, the imposition of decentralization has increased access to health services by increasing facilities and engaging communities (13–15). A perception of a decline in the availability and quality of services after decentralization implementation has been reported in Nigeria and Sudan. In contrast, Indonesia reflects a perception of patient satisfaction due to its decentralized authority in planning and management (3, 16–18, 37).

The significance of paying close attention to and comprehending the implementation phase of decentralization and its implication cannot be emphasized enough, as it is vital for attaining the intended outcomes (13–15). Nonetheless, implementation is often deemed the weakest link in policy reforms, particularly in developing countries (19). Despite being overlooked in policy design and planning, political considerations typically arise during practical implementation (11, 20). Additionally, various challenges can hinder the implementation process, including conflicting political motives, resistance to change, fear of losing authority, and inadequate planning, all of which significantly affect the final outcome (21, 22). Exploring and understanding the implementation phase of decentralization and its implications is essential for achieving the desired outcomes (13–15). Much literature has emphasized the crucial role of the implementation process in determining the policy’s success or failure. However, and to the best of our knowledge, little literature has examined the process of decentralization implementation and how it has impacted or shaped the policy consequences or outcomes. Documenting and examining this process in the local context, Khartoum State, Sudan, from the perspectives of healthcare providers and policymakers, using a conceptual framework, is crucial. In addition, this documentation will enable the identification of strategies necessary to maximize the impact of decentralization and plans for post-war state building.

## Methods

### Study design

This was an exploratory qualitative study that employed semi-structured observations, in-depth interviews, and thematic analysis. The study was conducted from July to December 2015 and involved policymakers and healthcare providers from the four hospitals that underwent decentralization in 2012 in Khartoum state. The study evolved around documenting the entire process from its early stages of decision-making, policy formulation, and implementation while also examining the influence of contextual factors.

### General setting

Sudan is a low- and middle-income country that experienced several internal armed conflicts since its independence in 1956. The country has a population of 46,874,204 million, with approximately 66% residing in rural areas. Sudan’s poverty rate is at 66.1% in 2022, and it carries an external debt burden of approximately $56 billion [163% of gross domestic product (GDP)] (23). Disparities exist in the distribution of health resources among Sudan’s states, with Khartoum and Gazira states hosting 27% of the country’s public hospitals, 30.5% of private facilities, and 25% of primary healthcare (PHC) facilities (24). Moreover, approximately 70% of the healthcare workers are concentrated in urban areas (25). As part of broader economic and political liberalization policies, the country underwent territorial division into 18 states. It adopted a structural adjustment program (SAP), which led to budget cuts in healthcare and other social services (38).

### Specific setting

The study was conducted in the Khartoum locality, one of the seven localities that make up the Khartoum state. The population in this locality consisted of urban, rural, semi-urban, and internally displaced populations. It comprises 13 public hospitals (including three secondary multispecialty hospitals and 10 tertiary single specialty hospitals), 31 public primary healthcare centers (PHCs), 10 non-governmental organization (NGO) health centers, 60 health centers, 98 private hospitals, and 601 private clinics (24). Khartoum locality has been selected due to the significant implementation of decentralization compared to other localities. The forms of decentralization were transference from certain hospitals to smaller peripheral ones, as described in a chronological form in Boxes 1–3.

BOX 1: The history of decentralization in Sudan (1951-1968)

Overall decentralization during colonialism:

- Imposed initially through the participation of local community leaders in administrative matters.
- Establishment of district councils in 1951, funded by local communities’ contributions.
- Poor coordination, insufficient financial resources, and underfunding of services at the district level were the main challenges (26).

Overall decentralization after independence (1956-1968)

- Provincial authorities were established in each province after a military coup in 1958 with financial means to support service delivery.
- The local structures continued to operate until the public revolutionary movement took power in 1964.
- After 1964 decentralization was challenged by a lack of clear organization and of financial and management capacities [Elabbasi, 2003; (26)]. Not considering lessons learnt and transfer of authorities without assessment of local capabilities were also major challenges (26).

BOX 2: The history of decentralization of healthcare services in Sudan (1969–1988)

- In the 1970s, a decentralization act and the imposed transfer of health services of the 1970s were part of the implementation of the structural adjustment program.
- A new regional governmental Act that devolves authorities to regions with more local autonomy, was introduced in 1981 in replacement of the 1970 act (27).
- Additional functions to regions, such as education, primary healthcare (dispensaries), drainage and water supply, agriculture, culture, and sports were assigned in 1981.
- Members of local structures were mainly selected by the ruling regime, while few were elected.
- Challenges such as overlapping responsibilities and insufficient resources despite taxation budgets persisted. Moreover, poor planning and guidance and lack of capacities at the local level led to difficulties in service delivery and fueled conflicts with central levels (28, 29), p. 65.
- After the public uprising in 1985, the same decentralized system continued, with representatives in regions selected from political parties in the government.

BOX 3: The history of decentralization in Sudan (1989-2012)

- The short-lived democratic structure was terminated by an Islamist military coup in 1989 (40). The coup replaced the representatives by member from armed forces and imposed a new local structures of public committees at the village level to maintain the regime's structure and deliver public services (30).
- Subsequently, the nine regions were divided into twenty-six states (40), and the 1998 constitution further solidified health system decentralization by assigning tertiary and secondary service to states and primary services to localities while reserving federal roles for policymaking and planning.
- The 26 states were later reduced to 16 and then 18 after South Sudan's separation, however, the federal level-maintained control over state duties through resource allocation.
- In 2010, a policy of full authority for secondary and tertiary health service delivery to be transferred from the Federal Ministry of Health to the state and locality level was imposed.
- Subsequently, in 2012, the Khartoum State Ministry of Health extended the policy which was a devolution of health services from central hospitals Khartoum Teaching Hospital “KTH” and Jafar Ibnoaf Child Health Hospital (JOH) to smaller district hospitals Ibrahim Malik Hospital “IMH” and Alacademy Hospital “AKH”.
- The implementation of decentralization in the health sector was significantly influenced by abrupt political internal and external factors rather than health-related considerations (40).
- Decentralization of health sector encountered resistance and strikes. Challenges, including inadequate financial and human resources, discrepancies between states and conflicts of interest between the federal and Khartoum state levels have also been reported (40).
- Efforts have been undertaken to stabilize the relationship between the federal government and the states, such as by providing financial incentives for human resource development at the state level. However, the situation remained unpredictable due to the imbalanced power relation between federal government and the states, particularly in Khartoum (40, 41).

### Theoretical framework

The literature explores several degrees and forms of decentralization. For example, Bossert and Beauvais (39) defined decentralization as the transfer of fiscal, administrative, ownership, and political responsibilities from central institutions, such as the Federal Ministry of Health, to local institutions in response to the health needs of local communities. However, decentralization takes on multiple forms, and this paper specifically focuses on four types: de-concentration, delegation, devolution, and privatization, as described in Table 1.

**Table 1:** Key concepts and definitions of decentralization.

Type of decentralization	Description
De-concentration	Transfer of administrative responsibilities from the district-level offices but within the central or federal structure. It is the least radical form as it only encompasses administrative roles
Delegation	Transfer of management responsibilities from central government to external institutions or organizations indirectly controlled by the government. These external entities usually have their funding, management staff, and legal frameworks.
Devolution	Transfer of political, administrative, fiscal, and ownership responsibilities of facilities to sub-national levels. These levels operate independently from national, with clearly defined geographical boundaries, legal status, and access to revenues and expenditures. It represents the most radical form.
Privatization	Transfer of government functions, specifically health service delivery, to voluntary organizations or private profit-making or nonprofit-making organizations

*Source (22).

**Figure 1:**
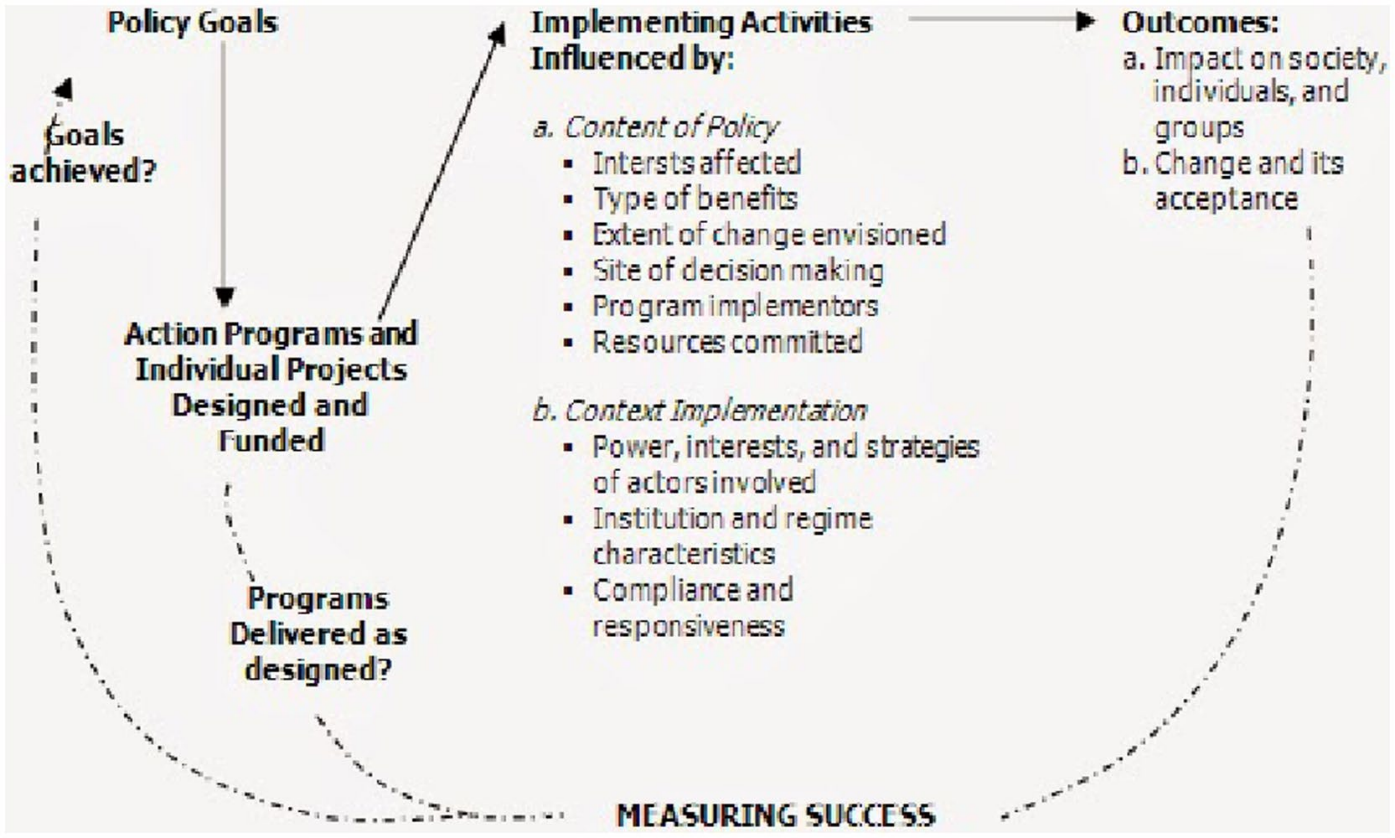
Implementation model as a politico-administrative process (11).

Generally, implementation is defined as a process that involves the execution of specific programs and projects that translate policy from theory into practice. This study employs Grindle’s (11) analytical framework for evaluating public policy implementation and portrays the implementation process as a multifaceted social and political process rather than a straightforward execution of policy directives (Figure 1). Consistent with Grindle’s argument that policy implementation not only influences policy outcomes but also is shaped by them, and can ultimately impact policy contents, consequences, and long-term effects, implementation is regarded as the most vital step in the policymaking process. In many developing countries, policies are often implemented without considering the characteristics of the political regimes in which they operate. Inadequate planning and resource allocation for implementation result in policies failing to achieve their objectives (11).

Grindle’s model underlines the significance of both the policy content and implementation context in affecting the decision-making process and final outcomes. It emphasizes how the program’s content and dynamics between decision-makers in a particular context impact the decision-making process. Moreover, it acknowledges the potential for challenges in implementation to result in adjustments to policy goals or reinterpretations of policy content and goals based on the feedback received during implementation (Figure 1). Additionally, the content of a policy can influence its implementation. Clear goals and objectives, along with a shared understanding of policy goals among the actors, can facilitate implementation. Policies that challenge power and resource distribution, such as decentralization, often encounter resistance. Managerial capabilities can also hinder delegations.

The implementation context encompasses the nature of the political regimen, the power dynamics between actors and their interests, and the outcomes of other programs and policies. Consequently, similar programs can be implemented in different ways depending on the context.

### Selection and description of study participants

For this qualitative study, participants were randomly selected from the list of health workers at two referral hospitals from which services were transferred [Khartoum Teaching Hospital (KTH) and Jafar Ibn Oaf Hospital (JOH)], as well as from two district hospitals that received the transferred services [Ibrahim Malik Hospital (IMH) and Alakademy Hospital (AKH)] in Khartoum State. A total of 69 health workers (including 30 medical doctors, four nurses, two midwives, seven lab technicians, seven pharmacists and two assistant pharmacists, four statisticians, and nine administrative staff) were recruited out of 118, 474, 186, and 250 from the KTH, IBHM, JOH, and AKH hospitals, respectively (Table 2).

**Table 2:** Characteristics of study participants in Khartoum locality, Sudan.

Characteristics	Number
Sex	
Male	34
Female	35
Profession	
Doctors	30
Nurses	4
Pharmacists	7
Assistant Pharmacist	2
Lab technician	7
Administrative staff	9
Statisticians	4
Midwives	2
Policy maker at KMOH	1
Policy maker at FMOH	1
Facility level	
Tertiary hospitals	
KTH	19
JOH	12
Secondary hospitals	
IMH	20
AKH	18

Participants received both written and verbal information about the aims of the study. The signing of the informed consent form was required from all participants. Saturation was determined when no new knowledge or information emerged during the interview.

### Data collection and entry

Data were collected through semi-structured observations and in-depth interviews using a pretested topic guide that covered the perceptions of study participants about the content of the policy, the decision-making process, the involvement of stakeholders, and whether they received orientation or training about decentralization. Moreover, the study participants’ experiences regarding the implementation and actual transference of the authorities and services.

The interviews were one-to-one and audio-recorded, each of them lasting approximately 1 h. The interviews were conducted in Arabic, and the transcripts were retranslated to English. Confidentiality and data quality were assured all through, and the data collection processes were peer-reviewed to verify quality and consistency daily and to streamline the procedure to enhance the credibility of results. Prolonged engagement in the field was ensured to build trust with study participants and to encourage them to reflect on their thoughts and perceptions regarding the different stages of the decentralization implementation. Peer review and evaluation of the interview process on a daily basis were used to check the validity and reliability of the study findings and the role of the researcher during the interview process, as well as to identify newly emerged study guides.

The participants were interviewed through certain guides to share their opinions, views, and personal experiences about the decentralization implemented in healthcare facilities as per the following topics:

- Perceptions regarding the decision-making process and policy content.
- Experience in the implementation process.
- Perception of the orientation or training provided about the decentralization of health services, if any.
- Experience and perspectives regarding the process of transference of services from the federal to the state level and from central to peripheral facilities, including strengths and weaknesses.

### Data analysis

Thematic content analysis guided by the theoretical framework was employed. A thorough reading of the transcript data was conducted multiple times to gain a comprehensive understanding of the context and data in an inductive approach. This was followed by manual coding, categorization, and theme generation, with cross-checking to ensure accuracy. Participant anonymity was preserved through de-identification and the removal of all personal information that could be linked to specific individuals. The researcher’s knowledge of the context and cultural background of the participants helped build trust and enhanced the credibility of the findings. Furthermore, the triangulation of in-depth interview and observation data further strengthened the credibility of the study results.

### Ethical consideration

Ethical clearance was obtained from the National Research Ethics Review Committee at the Federal Ministry of Health (FMOH) in 2015, the Regional Committee for Medical-Health Research Ethics (REK) in Norway, and the Norwegian Social Science Data Services (NSD-44106/3/LB). The research objectives were clearly explained to all participants, and an information sheet was provided. The information sheet included details about the study scope, major themes, anticipated interview duration, voluntary participation, and the option to skip questions or withdraw at any time. Signed informed consent was obtained from all participants before commencing the observation or interviews.

## Results

Of the 69 health workers who were randomly selected from the list of health workers of the four hospitals, 47 had work experience exceeding 5 years, making them witnesses to policy implementation. Among the health workers, 34 were males and 35 were females. Additionally, policymakers, one from the Khartoum State Ministry of Health and the other from the Federal Ministry of Health were recruited for this study.

Several themes emerged from the interviews and were classified following a sequence in alignment with the framework:

- Design of selected decentralization.
- Policy content and decision-making process.
- Context of decentralization implementation.

### Design, policy content, decision-making process of selected decentralization

Sudan’s initial attempt at implementing devolution took place during Numeri’s regime, which saw the release of the Local Government Act of 1971 see Box 1 (29). Subsequently, the devolution of health services was implemented through the establishment of health areas (40). The devolution process was initiated in Khartoum state in 2012, with the transfer of service delivery from the federal level to the state and from large hospitals to district small hospitals (see Box 1).

The decision to decentralize was based on a presidential decree, and it was described as political. The main observation and statement was the lack of formal documents to govern and guide the devolution of health. In addition, it was suggested that the decision was influenced by the desire to sell the land of the hospital or by the use of decentralization and service transfer to alleviate overcrowding in the central area caused by the presence of large hospitals and universities. Another motive mentioned was to promote private hospitals, and informants highlighted that the then State Minister of Health was involved in healthcare investment. “Transference of hospital ownership from the federal to state is a political decision taken by a senior politician” (Interview No. 63. 21/12/2015).

“The decentralization decision was not based on scientific evidence; it was a security-based decision that aimed to control the recurrent political movements and strikes among healthcare providers and centered historically in Khartoum teaching hospital” (Interview No. 34. 2/11/2015). Most study participants expressed that they were not involved in the decision-making process and were surprised by the decision to transfer them to peripheral facilities.

“As health workers we were not involved in the decision making even our administrative staffs were not involved” (Interview No. 3. 3/10/2015).

“The ministry has no vision; they did not tell us about any specific plan for transferring the department, to prepare ourselves. We keep hearing talks about transferring the surgery or orthopedic departments. I have no access to any information as a head of the department, and this is evidence for the absence of a clear vision in the ministry. There is no research on diseases before taking a decision of making the hospital a reference one” (Interview 47. 19/11/2015).

However, some participants mentioned attending a workshop organized by the Khartoum State Ministry of Health, where representatives from the FMOH and the health workers’ union were present. However, the purpose of the workshop seemed to be primarily to inform health workers about the decision to avoid objections rather than involving them in the decision-making process. Nonetheless, the study participants expressed a consensus that they were not adequately trained or informed about the decentralization of healthcare services. “There were no enlightening sessions or workshops conducted for health workers regarding the devolution process, leaving them unaware and uninformed” (Interview No. 32. 1/11/2015). “The problem is there were no informing session to the workers about devolution, but I used to hear it on television, no one came and told us anything, no training, no workshop about devolution for the health workers” (Interview No. 32. 1/11/2015).

Some participants from the Khartoum teaching hospitals were misinformed about the policy. “We have been believing that it did not involve service transfer and that the hospital would be improved to function as a referral hospital, as revealed in interviews with participants from Khartoum teaching hospitals” (Interview No. November 46, 25/11/2015).

“The health workers in the Obstetrics department were misinformed that the services will not be transformed from KTH; instead, the department will be improved to a big referral facility” (Interview 46. 25/11/2015).

The study participants emphasized that there were no written orders throughout the process, and decisions were made through verbal agreements due to fear of taking responsibility.

“Throughout the events of closing and transferring units, there were no written orders, but it was only a verbal agreement because they were afraid of taking responsibility” (Interview No. 46. 25/11/2015).

“Services were transferred without documentation or a formal letter from the Ministry of Health” (Interview 47. 19/11/2015).

Specifically, the study informant described the devolution decision and process as follows:

- Transfer of Authority from Federal to State level

The implementation of federal decentralization began in February 1994. “It started in 1994 with the requirement to transfer hospitals from the federal to the state level based on geographic location” (Interview No. 62, 18/12/2015). Despite the interim constitution of 2005 clearly defining the distribution of responsibilities between the federal and state levels, implementation was delayed until 2009.

A committee was formed within the Federal Ministry of Health to facilitate the transfer of hospitals to the state. The implementation process is divided into two stages.

Stage 1: Nine small hospitals specializing in only one area are transferred from the federal to the state level. State governors formed committees to receive these hospitals on 1 January 2010.

Stage 2: Although Stage 2 started a year later with the formation of a joint committee between the federal and state ministries of health, the decree to transfer an additional nine larger federal hospitals was delayed until 29 July 2011. The Khartoum State Ministry of Health established a committee to receive these transferred hospitals, which officially came under the authority of the Khartoum State on 10 October 2011. Before executing the transfer, these committees assessed the human and financial resources, debts, and ongoing projects of the hospitals.

“We started with the implementation of the decree by launching a committee to identify health facilities included in the decree and its human and financial resources, debts, projects, and so on. According to the committee’s report, a Presidential Decree has been initiated to transfer ownership of these facilities to the state of Khartoum” (Interview No. 67. 21/12/2015).

- Transference of Health Services to Peripheries:

A committee was developed to assess the readiness of peripheral facilities to receive transfer services. Participants expressed concerns about the lack of preparedness for peripheral facilities when the services were transferred to them. “The services were transferred before these facilities were adequately equipped to receive them. Additionally, a committee was formed to assess the readiness of peripheral facilities before implementation, but the decision was overruled by the Khartoum Ministry of Health” (Interview No. 28. 28/10/2015).

It is worth mentioning that the process of decision-making and its implementation interlinks to the ongoing context, which finally shapes the outcome.

### Context of decentralization implementation

Considering the application of devolution and its implementation process, the interviews revealed that few participants, namely, those involved in child healthcare committees, were included in the implementation process. However, most participants were not involved in the planning or implementation of devolution.

The study participants shared their thoughts and opinions on how decentralization was carried out in terms of the distribution of resources, such as facilities, finances, and human resources, through a “top–down” approach. The results revealed a substantial disparity in the distribution of health facilities, which was believed to be a continuation of the colonial era. This disparity persisted throughout the expansion and decentralization of facilities. This disparity in the distribution of health facilities was identified by the participants as a contextual factor impacting the implementation of decentralization. Furthermore, ownership transfer from the federal to the state based on geographical location also doubled this burden of disparity. “The total number of devolved hospitals was 23, from which 18 hospitals were located in Khartoum state, two in the Nile River state, two in Gazira state, and one in North Kordofan state” (Interview No. 66. 22/12/2015).

“The misdistribution of facilities and health staff between the center and peripheries led to citizens from other states to depend on their health services in the center, especially secondary and tertiary level care” (Interview No. 63. 25/12/2015).

Other contextual issues that influenced decentralization implementation were the ongoing shortage of healthcare providers due to migration caused by poor salaries, lack of permanent job opportunities, and inadequate training and professional development; “I do not feel comfortable with my work; this country has nothing, if I had money I would have left it, I have been working for 5 years, and I have not had any course or training. It is not acceptable to write to the patient that he has to buy sutures for the catheter, and now the water supply is off in the nephrology unit, and we might not work on the next shift” (Interview No. 33. 2/11/2015).

“The salary is weak, and it’s not even enough for transportation, I’ve been working for 41 years, and I get paid only 1,200 SDG, that means 40 SDG. If I told you how much I pay for transportation and breakfast and clothes you will find out that I am working for free, and it’s been 4 months without us getting paid our incentive” (Interview No. 35. 4/11/2015).

Moreover, the political motivation that resulted in the withdrawal of hiring new healthcare providers, which exacerbated the shortage of staff in health facilities, was also reported. “I applied to the ministry 4 or 5 times, and there are no permanent jobs, only temporary contracts, having no job is good for me, when it gives me the opportunity to travel abroad so they will not hold me especially that one is willing to leave” (Interview No. 33. 2/11/2015).

“In Ibrahim Malik Hospital is full of junior doctors because senior doctors left the country to work abroad after the closure of Khartoum hospital” (Interview No. 1. 2/10/2015).

These discrepancies and inequitable distribution of facilities and human resources for health between the center and peripheries are a cause of inaccessibility and greater reliance on the population of peripheries at the center, particularly for secondary and tertiary services. “About 70% of regular patients in the GIT center are from outside Khartoum state” (Interview No. 63. 25/12/2015). The services provided in federal health facilities before decentralization were described as comprehensive, as exemplified by the Khartoum Hospital, where various departments and specialties were in one place. Patients can easily access multiple services within their premises, including medicine, surgery, nephrology, trauma, and diagnostic procedures. “In my opinion, everyone who gets sick is better off going to Khartoum hospital, where he/she can find comprehensive services. Now after devolution, they have stopped Khartoum hospital, except for the surgery department which will be shut down in the coming days” (Interview No. 4. 3/10/2015).

The study participants noted that decentralization was expected to decrease the gap between the center and peripheries in terms of the availability of equipped facilities and specialized services. However, the lack of planning and coordination to ensure smooth equipment has augmented the existing disparity and burdened unready peripheral facilities. The implementation was not well prepared, mainly in terms of readiness of the peripheral setting to receive the transfer. “We know there are services that will be provided, although there is a difference in the capacity that we can receive (and) the number of patients coming. For example, at the surgery (section/department) there were 50 beds, 25 for men, 25 for women, so when they brought in new units (departments) and we received an excessive number of patients, for example about 100 extra patients, where would they go?” (Interview No. 58. 16/11/2015).

“I noticed that all the emergency examination rooms were narrow and badly ventilated due to the lack of vents. Also, the bridge joining between the emergency and the operating room is in the pier area, an area that is sinking into the ground due to the inability (of the ground) to withstand the bridge’s weight, due to the presence of groundwater. And sewage remains inside (beneath) the emergency section building which was closed because (the sewers) constantly overflowed” [Observation Note: (No. 6) 26/11/2015]. “During my presence in the Obstetrics and Gynecology department in Alacademy hospital, healthcare workers complained of the recurrent cut off of water supply and at the same time power cuts, many times although the operation room was occupied at that time” [Observation Note: (No. 2) 16/10/2015].

Furthermore, some participants argued that there was no need to transfer services from federal facilities to district hospitals. Instead, they believed that services should have been improved in the peripheries. “They should have improved the peripheries by establishing fully equipped facilities and enhancing the federal facilities to serve as tertiary facilities” (Interview No. 65. 20/12/2015). It was also perceived that the services were just transferred from one geographical central area to another, as Ibrahim Malik and Alacademy hospitals are both located in the center of Khartoum state.

Study participants strongly perceived a lack of planning and poor coordination in the implementation process. Consequently, life-saving services were denied to patients who arrived at Khartoum and Jafar Ibnoaf hospitals. For example, some patients in labor pain had to seek care at peripheral facilities after being unable to receive assistance at Khartoum Teaching Hospital. “Also, an associated closure of hospitals, primary healthcare centers, and departments within hospitals, such as Elbanjadid Hospital, Sanna Hospital, Saggana Primary Healthcare Center, and the Child Health Department in Haj Elsafi Hospital, located in the northern and eastern parts of Khartoum North, was noted as a consequence of decentralization implementation” (Interview No. 40. 17/11/2015).

“In Khartoum teaching hospital there was an emergency room for prioritization and sorting out of emergency cases, but it was closed during the process of the devolution implementation; so some cases of asthma were found died in their cars while waiting to see the doctor” (Interview No. 40. 17/11/2015). Furthermore, there were instances of reverse transference of health services during the implementation of decentralization. Instead of transferring services from large facilities to smaller ones, the opposite occurred. For example, the services were transferred from the Salamat Center, the only insurance center in the area, to Bashair District Hospital. “Sanna and Salamat hospitals were in service. Salamat was shut down, and the equipment in it had been transferred to Bashair hospital, despite it being an insurance center” (Interview No. 3. 3/10/2015).

“Additionally, the dentistry service was moved from Sameer Primary Healthcare Center to Alacademy Hospital to serve the interests of the Minister of Health, as the minister’s university students were trained at Alacademy Hospital” (Interview No. 40. 17/11/2015).

The context of service transfers had its features in the case of child-related health care services. Our data revealed that the transference of child health services from Jafar Ibnoaf Hospital occurred in three subsequent ways in 2012. First, the delivery of emergency services was suspended without the involvement of the community. However, patients continued to seek emergency services at the hospital, leading to a critical situation. The health workers at the hospital raised this issue with the hospital administration, as they recognized the urgent need for emergency intervention. A compromise was reached through the establishment of a section called “day one” to handle emergency cases. Unfortunately, this intervention failed due to a shortage of equipment and trained staff. Second, several departments were demolished. “Some departments, Ward C15, the Hematology unit, the Malnutrition treatment wards, the isolation wards, the Medical Periscope department, and administrative offices” (Interview No. 40. 17/11/2015). Also, hospital beds were transferred to Bashair and Ibrahim Malik hospitals in an unstructured way that lacked documentation, and formal notifications raised concerns of participants. “Due to the lack of coordination, official documentation, and structured handover procedures during the equipment and machine transfer, lab equipment’s, 14 ICU beds, 25 incubators, X-ray machines, and a central cooling system went missing during the decentralization implementation process” (Interview No. 40. 17/11/2015).

Resistance from healthcare providers emerged when the Ministry of Health attempted to transfer the general child and echo-cardiography departments. The former was to be transferred to peripheral facilities, while the latter was to be transferred to Alshaab Hospital for chest and cardiac services. As a result of this resistance, the ministry only transferred the pediatric cardiology specialist to Alshaab Hospital while keeping the equipment at Jafar Ibnoaf. However, the pediatric cardiologist continued to provide services at Jafar Ibnoaf Hospital.

Third, in October 2015, the Khartoum Ministry of Health awarded the emergency building of Jafar Ibnoaf Hospital to an NGO called Bint Al Balad for use as a child health oncology hospital. “The decision of giving emergency building to Bint Al Balad NGO was made abruptly without involving the hospital administration or staff” (Interview No. 39. 16/11/2015). Representatives from the KSMOH development department and the board of directors of the NGO requested that the health staff at Jafar Ibnoaf Hospital vacate the emergency building. This meant that the workers’ affairs office, social services office, warehouse workers, secretaries, district staff, physiotherapy and nutrition departments, health workers union office, and engineering unit would need to be transferred to the dermatology hospital, or they would be forcefully evacuated. (Interview No. 39. 16/11/2015). The study participants viewed this as a forceful move by the ministry and showed disrespect to the consultants and academics at the hospital. Healthcare providers resisted the evacuation of the emergency department, and the health workers union gave the ministry a 72-h ultimatum to halt the evacuation, threatening to initiate a strike, as revealed in interviews with study participants (Interview No. 37. 15/11/2015).

The transference of Khartoum Teaching Hospital (KTH) services began with the closure of the Department of Obstetrics and Gynecology in response to a health workers’ strike in 2012. The decision to close the department was made by the Khartoum State Ministry of Health (KSMOH) to improve and transform it into a larger complex. In the same year, the KSMOH attempted to close the department again, this time claiming that it was contaminated with bacteria, without conducting any tests or examinations. “The Department of Obstetrics and Gynecology was transferred because of the presence of bacteria, all workers transferred to the department to Ibrahim Malik hospital or another hospital like Alturki depending on their home” (Interview No. 62. 7/12/2015). The Ministry of Health tried to increase pressure on the department’s administration and staff by publicly disclosing the contamination in newspapers and other media outlets. Subsequently, the department was demolished by police officers and bulldozers, and the administrative offices were relocated to the internal wards in the gynecology and female surgery complex. In response to these actions, the hospital administration formed a committee headed by the head of the obstetrics and gynecology department, along with other specialists, representatives from the quality control department, a medical manager, a representative of the health workers’ union, and an assistant managing director. Samples were taken from the newborn department, which came out negative, but the Ministry of Health remained adamant about closing the department. Another committee was formed by the Ministry, but their recommendation was to reopen the obstetrics and gynecology department.

“The hospital administration constituted a committee headed by the head of obstetrics and gynecology department. And membership of other obstetrics and gynecology specialist (decision maker), two subspecialists, and representatives of: the quality control department, medical manager, health workers’ union, and assistant managing director” (Interview 46. 25/11/2015).

There were plans to demolish the morgue department and the department of physiotherapy, but resistance and demonstrations within the physiotherapy department prevented it. Instead, the KSMOH started transferring healthcare workers, beginning with the medicine department, urinary section, obstetrics and gynecology healthcare workers, and neurology department staff. These health workers were transferred to Ibrahim Malik Hospital.

The nephrology department was also targeted for transfer. Dialysis machines were moved during weekends and at night but faced resistance from health workers. The Ministry of Health responded by transferring and withholding the salaries of the health workers. To avoid further resistance, the ministry focused on transferring the nephrology department during vacations. Regular dialysis machines were forcefully taken to Alacademy Hospital, while emergency dialysis machines were transferred to Bahri Hospital. “The transfer of the renal unit from Khartoum Teaching Hospital was under gunfire” (Interview No. 53. 23/11/2015). Extreme violence was used during the transference of the psychology department, which supported patients with AIDS. Police officers broke into the office, confiscated equipment and tables, and even threw away patient files. This led to physical confrontations between the police and healthcare workers and demonstrations involving HIV/AIDS patients.

“The transference of psychology department involved hand fight between police and healthcare workers and demonstrations after that with the participation of some HIV/AIDS patients as perceived” (Interview 33. 2/11/2015).

The official closure of the obstetrics and gynecology department occurred after pressuring the universities of Khartoum and Alneelain to transfer their staff to peripheral facilities. Retired obstetrics and gynecology specialists were directed to work only in the fistula department.

“The ministry has no vision; they did not tell us about any specific plan for transferring the department, to prepare ourselves. We keep hearing talk about transferring the surgery or orthopedic departments. I have no access to any information as a head of the department, and this is evidence of the absence of a clear vision in the ministry. There is no research before deciding on making the hospital a reference one” (Interview No. 47. 25/11/2015). Moreover, there was a lack of systematic documentation, standard operations, and formal letters from the ministry to notify staff about transfers. Patients were also transferred without proper evaluation or screening, leading to convulsions and distress during the referrals, even for patients in the intensive care unit (ICU) (Interview No. 47. 25/11/2015). “Throughout the events of closing and transferring units, there were no written orders, but it was only a verbal agreement because they were afraid of taking responsibility” (Interview No. 46. 19/11/2015). Patients were not evaluated before transferring “They transferred all patients from Khartoum hospital even those in the ICU. They treat patients like cattle, but do not wait until the patient’s condition improves” (Interview No. 23. 28/10/2015). In addition, approximately 1,000 health workers were transferred from the KTH to peripheral facilities. This unsynchronized action caused mismatched specialty transfers. “Some healthcare workers were transferred to facilities where their specialties were not available, such as surgery consultants being transferred to Umbadda Hospital, which had no theater. Hematology specialists were also transferred to laboratories without a hematology department, as reported by the study participants” (Interview No. 48. 19/11/2015).

To address the resistance of health workers, the ministry outsourced the financial resources of the hospital by closing the Southern private unit and stopping entrance fees for co-patients. The authority of the pharmacy was transferred to the “Revolving fund pharmacies,” run by the Khartoum state drug supply. The hospital’s emergency services were gradually closed with shared duty schedules and patient transfers to other hospitals. The official closure of the emergency department was issued in October 2015. The ICU of the internist emergency department was closed, and patients were moved to the surgical complex ICU while the equipment was stored away. The operating room in the emergency department was destroyed due to allegations of bacterial contamination without any investigation or reports. Finally, on 31 December 2015, emergency services for all hospital departments were closed.

The department of surgery was transferred to other hospitals, and the Khartoum Teaching Hospital had only referred clinics for cold cases of pediatric surgery, urology, and a fistula department. More possibilities were discussed among hospital staff to preserve the service provision. “We thought of either transferring KTH to become a referral hospital for orthopedics or relocating the orthopedics department to the National Center for Orthopedics and Plastic Surgery. Another suggestion was to establish a pediatric surgery center within the hospital. However, most hospital staff believed that the hospital would be closed, as skilled staff, equipment, and departments such as urology, medicine, and nephrology had already been transferred, according to our study participants” (Interview No. 45. 19/11/2015).

The conflict over the ownership of national specialized centers was another prominent issue arising from the decentralization decree. The federal ministry and the Khartoum State Ministry of Health had conflicting views on whether these centers should be considered decentralized or remain under federal authority. Attempts were made to change the name of these centers from National to Khartoum state centers, but resistance from the center directors caused complications.

The conflict escalated, leading to the formation of a higher board of national centers and tertiary hospitals that aimed to evaluate the situation and make recommendations. “These recommendations faced resistance from the Khartoum State Ministry of Health, which believed that the centers should be financed by the federal ministry but operated under the state ministry’s authority” (Interview No. 67. 21/12/2015).

The privatization of health services was also highlighted as a context factor that contributed to the context of the implementation. Privatization had initially started with non-medical services such as nutrition and cleaning but later extended to include medical services. “To cover expenses of services, the hospital offers cesarean section and natural labor by charging patients” (Interview No. 62. 7/12/2015).

“The delivered service in the national centers are also privatized. For example, in the neurology center admission to the ICU costs 5000SDG, although it is a public center, because the center has no allowance from the ministry of health, and the operational budget is collected from the price of the service” (Interview No. 63. 25/12/2015).

The decision to transfer services before ensuring the readiness of peripheral facilities and the claim that services were transferred to the peripheries was attributed to a private biased interest, as the Khartoum state minister is a private investor. “The unfortunate appointment of a private professor as a minister of health which was certainly a mishap and totally against the law due to conflict of interest. He started his work by the dismissal of existing committees and senior consultants in every field to avoid objection to his decisions” (Interview No. 40. 17/11/2015).

“Another motive for emptying the areas of the center from public hospitals is the advancement of private hospitals, as the minister of health from investors in the field of health” (Interview 34. 2/11/2015).

The study revealed the interlink between poor decision-making, insufficient planning, and the exclusion of health workers, the unaddressed disparities in facility readiness and preparedness, ultimately leading to a complex of consequences. The context of decentralization implementation added further complexity to the situation.

## Discussion

This study provided insights into the process of decentralization of health services implementation in Sudan, focusing on various stages of policy implementation, decision-making, and the actual implementation process. Grindle’s theoretical framework was employed, which emphasizes the conversion of policy goals into realized outcomes through the implementation process. It also highlights the influence of policy design, contextual factors, and implementation on policy content and outcomes. This discussion is organized according to Grindle’s framework, with the following sections:

### Design and content of the decentralization policy

#### Decision-making process

One key finding of this study was the consensus among participants’ perceptions that the decentralization decision was purely political and lacked a technical or scientific basis. It was driven by federal decentralization outlined in the 2005 Constitution, which assigned responsibilities between the federal and state levels. While the federal level handled policy formulation and planning, states were responsible for daily service delivery.

In 2012, only the KSMOH implemented decentralization of health services in Khartoum state without monitoring from the federal level or higher authorities. This underlines a possible environment of alienation for the main player, which might affect the ownership of the decision.

Moreover, inclusiveness in decision-making and managing health service delivery, including citizen participation in prioritization, planning, resource allocation, and monitoring, was not given due attention. The early involvement of the designated association would have supported readiness and preparedness in the practical execution of devolution in a well-phased manner. The lack of technical preparedness and capacity at the state level has severely undermined health service delivery, leading to the discontinuation or unavailability of essential services, such as emergency dialysis (18). This fragmentation of services has compromised their overall quality. Consequently, many health service users have been forced to seek care in the private sector, which, in turn, could further empower the private sector (24). An assessment has also revealed a challenging relationship between the federal and provincial levels and highlighted the need to strengthen the structure and the capacities on different levels (31).

The decentralization decree was not discussed conceptually by healthcare leaders, which augmented the perception that this presidential decree is political and has been enforced, regardless of the stakeholders’ perception. This has also raised the sense that there might be a hidden agenda from decentralization. Opinions expressed in the interviews indicated that hidden aims, such as privatization of health services and selling hospital land, guided the implementation process. Similar findings were observed in Nicaragua, where decentralization through de-concentration was followed by devolution, which was different from the experience of Sudan in terms of gradual transference of the authorities. Moreover, the similarity with Sudan’s experiences in the sociopolitical context in which decentralization was implemented as a requirement from the World Bank and in the context of structural adjustment programs and a free-market economy (21). In such cases, decentralization could become a means of promoting free-market principles within the health sector, as observed in Nicaragua.

The decentralization masked the implementation of structural adjustment programs in the health sector (4, 21). Subsequently, resistance to the decentralization decree was observed among healthcare providers, leading to strikes and demonstrations in certain hospitals faced with violence from the Ministry of Health. This resistance to decentralization has also been observed in other countries such as the Philippines, Botswana, Papua New Guinea, and Nicaragua (4, 21, 22). In the Philippines, decentralization by devolution was introduced in a context marked by disparities in the distribution of healthcare facilities, a challenge the policy failed to adequately address (4). This experience was similar to the Sudan experience in terms of the degree of the authorities’ transference, the abrupt implementation of the policy, and the contextual disparities regarding the geographical distribution of health facilities. In Pakistan, decentralization, in the form of devolution, was implemented following a political revolution aiming at meeting the decentralized demands in leading an equitable contributory social sector (32). A similar abrupt enforcement of decentralization following a revolutionary change was also seen in Indonesia (33). The experiences of Pakistan and Indonesia were similar to Sudan’s experiences regarding the implementation of the same degree of authority transference as in Pakistan. Furthermore, the implementation of decentralization of health services is a response to the broader political decentralization in an abrupt way. In Papua New Guinea, decentralization by devolution was aimed at countering the highly centralized political system inherited from the colonial era, as well as addressing significant disparities in wealth distribution across geographic regions (42). Many similarities were identified between the Sudan and Papua New Guinea experiences in terms of the degree of authority transference.

#### Stakeholder involvement

The research also revealed a lack of transparency and a top–down approach to implementation, with little stakeholder involvement. This approach is common in highly authoritarian (43). Legitimacy, government stability, potential opposition from influential interest groups, and the position of the elite are all political resources that influence policy implementation. Power differentials among actors, their interests, and the characteristics of the regime within which they interact shape the implementation process (43).

This little stakeholder involvement in the decision-making and implementation of decentralization of health services can be attributed to the political nature of the regime and its underlying motives. The primary aim of decentralization in this context was to build political legitimacy and consolidate patronage networks (28). From 1989, professional trade unions, as hubs for possible stakeholder engagement, were dismantled, and regime-appointed bodies were replaced. Additionally, the conflicts of interest among those implementing the policy might have further contributed to the lack of stakeholder involvement.

This study highlights the political nature of the decision, resistance among healthcare providers, lack of stakeholder involvement, and the need for transparent and inclusive implementation processes. It also outlined the crucial prerequisites for the successful implementation of decentralization, such as the availability of financial and human resources, understanding of power dynamics, political and administrative resources, and the overall feasibility of the policy. These factors have been emphasized in the literature (29) and are essential for ensuring the effectiveness of the decentralization process.

Moreover, the overall political context in which decentralization took place in Sudan was under a military–coup–led regime. This might also have implications, and the regime aimed to legitimize its hold on power through federal decentralization, but without the inclusion of democratic processes and citizen participation (11, 20). Similar patterns have been observed in other countries such as the Philippines, Botswana, Nicaragua, and Papua New Guinea, where decentralized decision-making and implementation occurred without the involvement of stakeholders. In the Philippines, stakeholders are not adequately informed about service transfers, leading to resistance and demonstrations (22, 39, 44). Similarly, decentralization in Sudan lacked agreement with the National Congress and did not undergo any debate (41).

This study identified the use of misinformation and allegations, such as the contamination of hospital departments, as tactics to implement decentralization. Media and rumors were employed to exert pressure on health workers and to force their compliance with service transfers. Violent implementation of health staff transfers has two significant disadvantages. First, it perpetuated a centralized culture and structure at the local level despite the transfer of highly skilled staff. This echoes the literature that highlights the potential transfer of centralized structures and routines with staff transfers (39).

#### Extent of changes envisioned (the extent of authority transference)

The distribution of responsibilities in Sudan differed from other countries, such as the Philippines, where federal and state levels shared responsibilities for specialized services, human resource management, and drug supply (4). In Sudan, all these responsibilities were transferred to the state level, as noted by the study participants and supported by FMOH documents (41). The absence of clear definitions, assessments of capacity, and coordination mechanisms between different levels has led to conflicts of interest, such as in medicine control regulations. These issues have also been identified as problematic in other decentralization implementation processes (4, 32). It was evident that insufficient participation impacted the democratic objective of decentralization. Furthermore, the implementation created disparities and gaps, which could have significant consequences for access. The resulting inaccessibility might impose additional strain on communities, lead to severe consequences, and foster skepticism towards state institutions (9, 34).

### Context of decentralization implementation

#### Conflict of interest

Conflict of interest between the federal and Khartoum state levels was evident in the authority over tertiary facilities and specialized services, which were transferred to the state level. Furthermore, decentralization hindered the ability of the Federal Ministry of Health (FMOH) to fulfill its role in interventions during epidemics, emergencies, and expanding services and training. The state-level authorities had jurisdiction over health workers and institutions, but a clear mechanism for coordination between the different levels was absent.

The study’s strengths lie in its inclusion of a large number of study participants which allowed for triangulation of data from various perspectives and professional backgrounds. The use of multiple data collection methods, such as semi-structured observations and in-depth interviews, further enhanced the credibility of the findings.

The study’s limitations arise from the inherent subjectivity of qualitative methodology, which seeks to capture the varied perceptions and experiences of healthcare providers during the decentralization process in Sudan. Although this approach yields valuable insights, it restricts the generalizability of the findings. However, the inclusion of a large number of participants, representing different categories of healthcare providers and policymakers, allowed the study to capture diverse perspectives on the implementation of decentralization. These shared experiences and insights offer important lessons for future decentralization efforts in low- and middle-income countries with similar political and economic contexts and comparable levels of decentralization.

## Conclusion and implications

This study thoroughly documents the decentralization of the health services process in Sudan, highlighting key aspects. It reveals the political nature of decision-making, characterized by a top–down approach and lack of stakeholder involvement. Transparency, official documentation, and proper handover procedures were notably absent. This process occurred within the context of structural adjustment programs that had already empowered the private sector in Sudan.

Theoretically, decentralization aims to improve service delivery efficiency. However, its implementation was influenced by various factors. Discrepancies in the distribution of health facilities, human resources, and financial resources played a significant role in reproducing existing inequalities. Political agendas, such as maintaining the current regime, have historically influenced Sudan’s decentralization. These contextual factors shape the decentralization process, policy content, and its outcomes.

Reforming the decentralization policy is recommended. Given the current resource distribution disparities and the need to devolve financial, political, administrative, and technical responsibilities from the federal to state levels, a concurrent responsibility paradigm is suggested. This would involve shared authority between federal and state levels, with financial resource distribution negotiated collaboratively. States would implement secondary-level plans and policies, while the federal government would evaluate these and continue to provide tertiary-level services.

## Data Availability

The datasets presented in this article are not readily available because Political sensitivity of the topic and this study is not categorized under any of the above categories. Requests to access the datasets should be directed to bsm16@mail.aub.edu.
